# Clinical Management of Diffuse Low-Grade Gliomas

**DOI:** 10.3390/cancers12103008

**Published:** 2020-10-16

**Authors:** Giuseppe Lombardi, Valeria Barresi, Antonella Castellano, Emeline Tabouret, Francesco Pasqualetti, Alessandro Salvalaggio, Giulia Cerretti, Mario Caccese, Marta Padovan, Vittorina Zagonel, Tamara Ius

**Affiliations:** 1 Department of Oncology, Oncology 1, Veneto Institute of oncology-IRCCS, 35128 Padova, Italy; giulia.cerretti@iov.veneto.it (G.C.); mario.caccese@iov.veneto.it (M.C.); marta.padovan@iov.veneto.it (M.P.); vittorina.zagonel@iov.veneto.it (V.Z.); 2 Department of Diagnostics and Public Health, Section of Pathology, University of Verona, 37129 Verona, Italy; valeria.barresi@univr.it; 3 Neuroradiology Unit, IRCCS San Raffaele Scientific Institute and Vita-Salute San Raffaele University, 20132 Milan, Italy; castellano.antonella@hsr.it; 4 Team 8 GlioMe, CNRS, INP, Inst Neurophysiopathol, Aix-Marseille University, 13005 Marseille, France; emeline.tabouret@ap-hm.fr; 5 Radiation Oncology, Pisa University Hospital, 56123 Pisa, Italy; f.pasqualetti@ao-pisa.toscana.it; 6 Department of Neuroscience, University of Padova, 35128 Padova, Italy; alessandro.salvalaggio@phd.unipd.it; 7 Padova Neuroscience Center (PNC), University of Padova, 35128 Padova, Italy; 8 Neurosurgery Unit, Department of Neurosciences, Santa Maria della Misericordia University Hospital, 33100 Udine, Italy; tamara.ius@asuiud.sanita.fvg.it

**Keywords:** diffuse low-grade gliomas, surgery, targeted therapy, radiotherapy, chemotherapy

## Abstract

**Simple Summary:**

Diffuse low-grade gliomas (LGG) are relatively uncommon primary brain cancers. In recent years, the molecular, diagnostic, and therapeutic approaches have evolved. *IDH (isocitrate dehydrogenase)* mutations can affect the great majority of these tumors with distinct genetic and clinical characteristics, carrying a more favorable prognosis compared with wild-type IDH. In patients with LGG, the most common manifestation is seizure and new neuroradiological tools are available to improve the diagnostic and therapeutic pathways. Surgical intervention is performed with the goal of maximum safe resection; postoperative chemoradiotherapy showed benefits in selected patients. New treatments based on molecular profiling, new small molecule and immunotherapy approaches could improve survival and quality of life. In this review, in order to identify the optimal clinical management of patients with LGG, we discuss the relevant biological and clinical characteristics, new therapeutic approaches, and future research directions for these tumors.

**Abstract:**

Diffuse low-grade gliomas (LGG) represent a heterogeneous group of primary brain tumors arising from supporting glial cells and usually affecting young adults. Advances in the knowledge of molecular profile of these tumors, including mutations in the isocitrate dehydrogenase genes, or 1p/19q codeletion, and in neuroradiological techniques have contributed to the diagnosis, prognostic stratification, and follow-up of these tumors. Optimal post-operative management of LGG is still controversial, though radiation therapy and chemotherapy remain the optimal treatments after surgical resection in selected patients. In this review, we report the most important and recent research on clinical and molecular features, new neuroradiological techniques, the different therapeutic modalities, and new opportunities for personalized targeted therapy and supportive care.

## 1. Introduction

Diffuse low-grade gliomas (LLG) can be defined as tumors probably derived from glial cells and showing infiltrative growth and an absence of histological features of malignancy. They account for approximately 20% of all primary brain tumors and involve about 20,000 persons per year in the USA [1,2]. The role of surgery has radically changed in the past several years, assuming a central role in LGG management. Indeed, maximal safe resection represents the first step in LGG workflow.

The median survival of patients affected by LGG is widely variable, ranging from 5.6 to 13.3 years, depending on several factors, such as extent of resection and molecular features, including *isocitrate dehydrogenase* (*IDH*) *1* and *2* mutations, and 1p19q codeletion. Due to the relevance of genetic features for the prognosis of LGGs, these have been integrated with histopathological characteristics in the latest World Health Organization (WHO) classification of tumors of the central nervous system [1].

Most patients with LGG present seizures, though the natural history of these neoplasia can include a pre-symptomatic phase due to their low proliferation index. In their last phase, LGG show malignant transformation to high-grade glioma (grade III or IV glioma) and worsening of clinical symptoms. Magnetic resonance imaging (MRI) is the gold standard for the initial diagnosis of LGG; however, novel neuroradiological techniques, also based on nuclear medicine, have been shown to contribute to the diagnosis and follow-up of these tumors. Due to their rarity and to the few prospective clinical trials, the optimal treatment of LGG remains controversial. 

Herein, we review the recent molecular, diagnostic, and therapeutic advances on LGG.

## 2. Histopathology and Molecular Features 

Diffuse low-grade gliomas were traditionally classified based on their histopathological aspect and subdivided into oligodendroglioma, diffuse astrocytoma, and oligoastrocytoma [3]. Oligodendroglioma was defined as a diffusely infiltrating tumor composed of cells morphologically resembling oligodendroglia (i.e., having round uniform nucleus and clear perinuclear halo), while diffuse astrocytoma was defined as a diffusely infiltrating astrocytoma characterized by a high degree of cellular differentiation and slow growth [3]. Finally, oligoastrocytoma represented a diffuse glioma composed of a mixture of two distinct neoplastic cell types morphologically resembling the tumor cells of oligodendroglioma or diffuse astrocytoma [3].

After the discovery that tumors with the same morphology may harbor different genetic alterations, and that such features have prognostic significance [4,5], gliomas were reclassified integrating genetics and histopathology [1].

According to the latest World Health Organization (WHO) Classification [1], diffuse LGGs include: oligodendroglioma *Isocitrate Dehydogenase (IDH)* mutant and 1p/19q codeleted; diffuse astrocytoma *IDH* mutant and diffuse astrocytoma *IDH* wild-type (wt). Among these, oligodendroglioma *IDH* mutant and 1p/19q codeleted carry the best prognosis, and *IDH*-wt astrocytoma the worst (Table 1) [6].

Therefore, the diagnosis of LGGs currently requires an assessment of the mutational status of *IDH1/2* and 1p/19q codeletion. In the event that molecular features cannot be assessed, diffuse LGGs can be classified, based solely on histopathology, into oligodendroglioma not otherwise specified (NOS), and diffuse astrocytoma, NOS [1].

**Figure 1:**
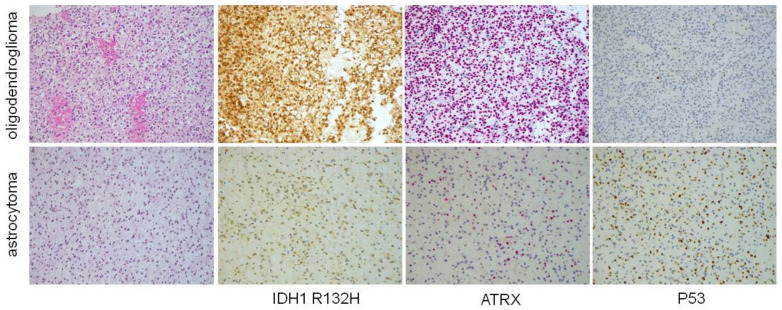
Morphological and immunohistochemical features of *IDH (isocitrate dehydrogenase)* mutant astrocytoma and oligodendroglioma (original magnification × 100). Oligodendroglioma is characterized by rounded monomorphic nuclei, while astrocytoma has oval nuclei and mild pleomorphism. Both these cases have *IDH1* R132H mutation, which is detectable by immunohistochemistry. However, astrocytoma has ATRX immunohistochemical loss and P53 diffuse staining as a consequence of mutations in these genes, while on the other hand oligodendroglioma, which lacks mutations in these genes, retains ATRX expression and is immuno-negative for P53.

About 90% of *IDH*-mutant diffuse gliomas have *IDH1* R132H mutation, which can be detected by immunohistochemistry using a specific antibody against the IDH1 R132H protein (Figure 1) [7]. A minority of cases have other (non-canonical) *IDH1* mutations at R132 residue (5%) [7] or *IDH2* mutations at 172 residue (5%) [5], the detection of which requires *IDH1/IDH2* sequencing.

Interestingly, *IDH2* mutations are mainly found in oligodendrogliomas, while *IDH1* mutations differing from R132H are mostly seen in astrocytomas [8,9].

The combined deletion of chromosomes 1p and 19q in oligodendrogliomas is mediated by a balanced whole-arm translocation of chromosomes 1 and 19, leading to the formation of two derivative chromosomes. One of these derivative chromosomes, being composed of 1p and 19q (der [1,19][p10; q10]), is typically lost [10]. 1p/19q codeletion can be assessed by several methods, including Fluorescent In Situ Hybridization (FISH) or Comparative Genomic Hybridization (CGH) [11]. FISH is the most widely used method, but, differently from CGH, it is unable to distinguish between whole arm deletion—which is specific to oligodendroglioma—and partial deletions—which can be also found in astrocytic tumors [11].

*IDH*-mutant diffuse astrocytomas commonly display inactivating mutations in *alpha-thalassemia/mental retardation syndrome X-linked (ATRX)* and missense mutation in *TP53* that are mutually exclusive with 1p/19q codeletion [12]. Since *ATRX* mutations result in protein loss and *TP53* mutations in p53 nuclear accumulation [12], a diffuse low-grade *IDH*-mutated glioma with astrocytic morphology, ATRX loss, and p53 diffuse and strong staining can be diagnosed as *IDH*-mutant diffuse astrocytoma in the absence of 1p/19q testing (Figure 1) [13] 

In the latest WHO Classification, the diagnosis of oligoastrocytoma is strongly discouraged and reserved to cases with ambiguous morphology when molecular tests cannot be performed (Oligoastrocytoma, NOS) or in the rare instance of dual-genotype oligoastrocytoma [1]. The latter is an *IDH* mutant tumor composed of two distinct populations, showing the morphological features and genotype of astrocytoma (*TP53* mutation/nuclear p53 accumulation, loss of nuclear ATRX expression and absence of 1p/19q codeletion) or oligodendroglioma (lack of TP53 mutation/nuclear p53 accumulation, retained nuclear ATRX expression and 1p/19 codeletion) [1,14].

WHO defines diffuse astrocytoma *IDH*-wt as “a diffusely infiltrating astrocytoma without mutations in the *IDH* genes” [1]. Therefore, this provisional entity is classified on the absence, rather than on the presence, of a molecular feature, and it likely includes genetically different tumors. In many cases, molecular analyses allow reclassification of *IDH*-wt diffuse astrocytoma into other tumor entities (i.e., pylocitic astrocytoma). Compared to *IDH*-mutated diffuse LGGs, *IDH*-wt ones involve older subjects and are less amenable to surgical resection [15]. In recent years, a great effort was made to define the molecular profile of these tumors (Table 1).

**Figure 2:**
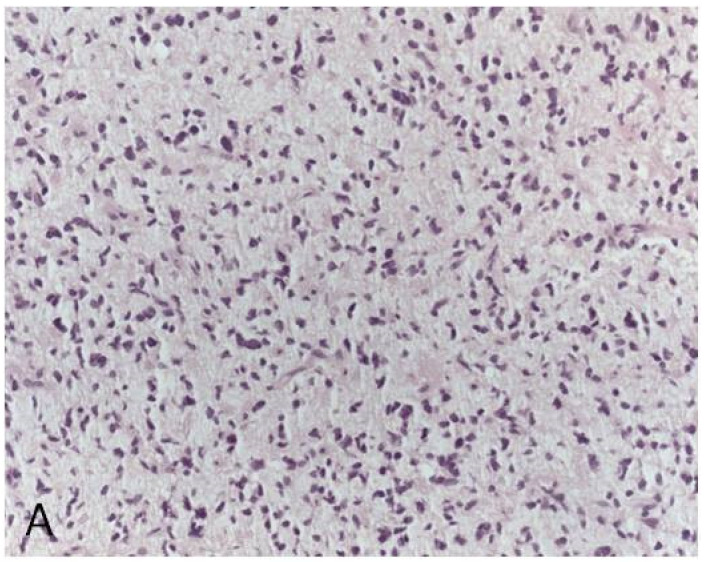

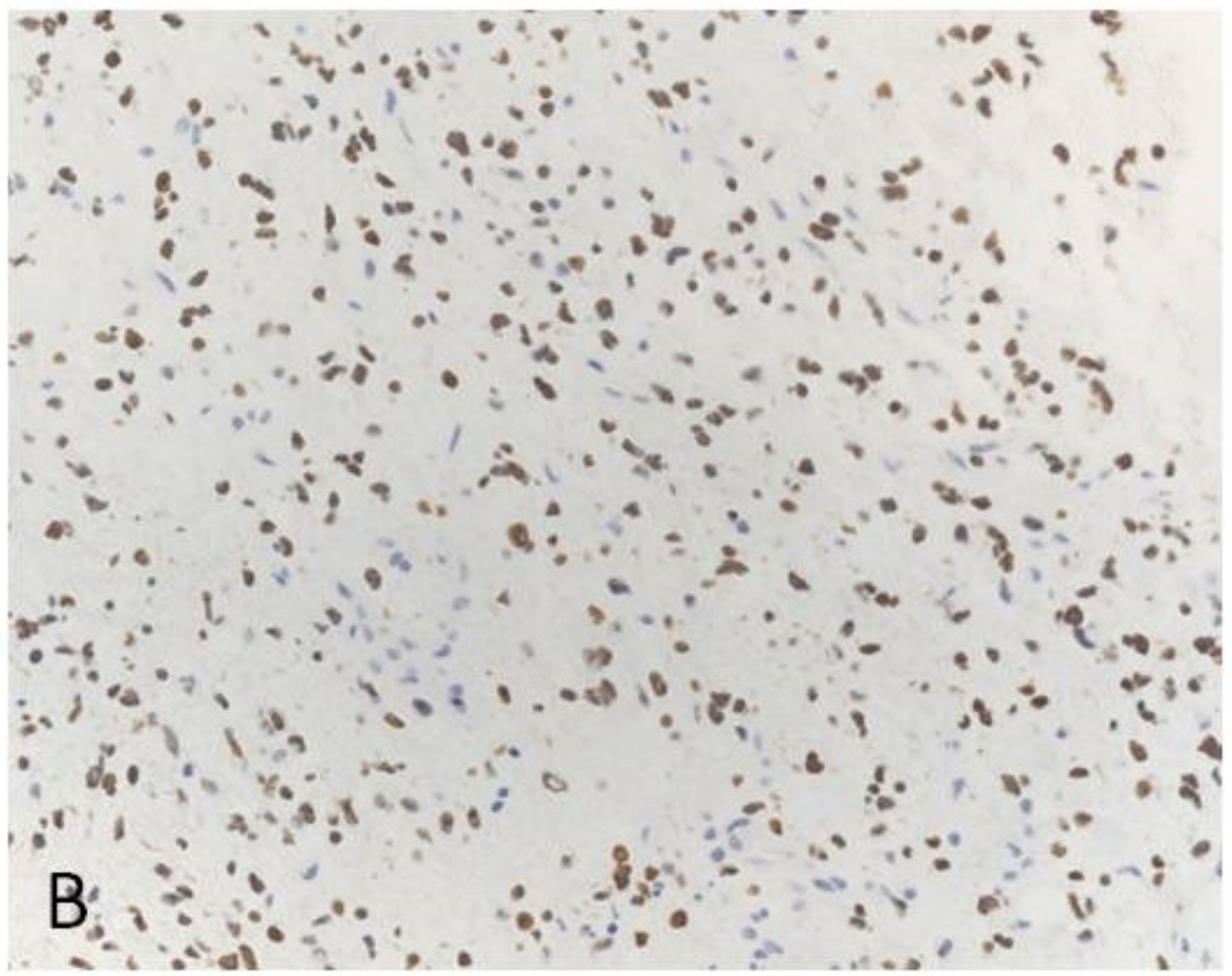
*H3* K27M mutated midline glioma (original magnification × 200). (**A**): Microscopic appearance with morphological features of a diffuse astrocytoma. (**B**): Nuclear immunohistochemical stain for H3 K27M protein.

A proportion of *IDH-wt* diffuse low-grade astrocytomas, located along midline anatomical structures (i.e., thalamus, pons, spinal cord, cerebellum), are characterized by mutation at position K27 in the histone genes *H3F3A* or *HIST1H3B* or *HIST1H3C* [1]. These tumors have significantly worse prognosis compared to midline gliomas without *H3* K27M mutation [16]. For this reason, they are considered to be a different entity, which was named diffuse midline glioma *H3 K27M*-mutated and classified as grade IV in the latest WHO classification [1]. *H3* K27M mutation can be detected by immunohistochemistry using an antibody specific to the mutated protein (Figure 2). 

A subgroup of *IDH*-wt/*H3*-wt diffuse LGGs in adults harbors a molecular profile similar to that of pediatric LGGs and consisting of *BRAF* V600E mutation, *MYB* or *MYBL1* structural variation, and *FGFR1* alterations (Table 1) [17]. Tumors with *BRAFv600E* mutation usually have astrocytic morphology, while those with *MYB*, *MYBL1,* or *FGFR1* alterations harbor oligodendroglioma-like histology [17]. 

The Consortium to Inform Molecular and Practical Approaches to CNS Tumor Taxonomy—The non-official WHO (cIMPACT-NOW) Working Committee recently suggested that these tumors should be classified separately from *IDH*-wt astrocytomas and termed diffuse glioma not elsewhere classified (NEC) [17].

Finally, some LGGs without *IDH* and H3 K27M mutations have the same genetic alterations as *IDH*-wt glioblastoma, i.e., gains in chromosome 7, losses in chromosome 10, focal amplifications in *EGFR*, *CDK4* and *MDM4,* focal deletions involving *CDKN2A* and *RB1,* mutations in *telomerase reverse transcriptase* (*TERT*) promoter [15,18]. They have only slightly better prognosis than *IDH*-wt glioblastoma. They probably represent early-stage glioblastomas [18,19].

## 3. Imaging

Magnetic Resonance Imaging (MRI) is the optimal neuroradiological technique for the study of LGG [20,21]. Conventional MRI (cMRI) provides an assessment of the morphological features of the lesion and the relationship with the surrounding structures. A standardized cMRI acquisition protocol has been recently recommended by the EORTC-NBTS consensus [22] and endorsed by the European Society of Neuroradiology (ESNR) [23] to be employed in the diagnosis and clinical management of lower-grade gliomas. By assessing the T2/fluid-attenuated inversion-recovery (FLAIR) abnormality and the possible enhancement on post-contrast T1-weighted images, cMRI is necessary for the initial characterization of a lesion as a possible LGG [20]. Susceptibility-weighted imaging (SWI) may contribute to identifying intralesional hemorrhage, calcification, or tumoral neovascularity, by detecting intratumoral susceptibility signal (ITSS) [24,25]. cMRI is also advised within 48 h from surgery to assess the extent of resection (EOR), which is among the most relevant prognostic factors for LGG [26].

In further evaluation of a suspected LGG or during treatment monitoring, serial contrast-enhanced MRI may identify new areas of contrast enhancement or significant changes in tumor size, suggesting malignant transformation [27,28]. Additionally, tumor size measurement using either 2D-FLAIR diameters or three-dimensional FLAIR (3D-FLAIR) volumetry has been shown to increase diagnostic accuracy in LGG follow up, the growth-rate being an early predictor of malignant transformation [28,29]. After surgical resection, volumetric tumor measurements may reflect clinical response better than 2D size changes, as they more accurately assess the extension of irregularly shaped residual lesions and the measurement is less affected by the presence of a previous surgical cavity. 

Along with cMRI, advanced MRI (aMRI) techniques, such as diffusion magnetic resonance imaging (dMRI), perfusion-weighted imaging (PWI), and magnetic resonance spectroscopy (MRS), add relevant structural, hemodynamic, and physiological information for tumor diagnosis and classification, surgical planning, and evaluation of treatment response [30]. By reflecting and quantifying the biological behavior and spatial and temporal heterogeneity of the tumor tissue, aMRI also provides new insights in characterizing molecular profiles of lower-grade gliomas by radiomics and radiogenomics [31,32,33]. Radiomics is the current state of the art in imaging analysis by extracting multiple quantitative imaging features from MR images in an objective and reproducible form, and represents the basis of radiogenomics, which aims at determining the association between quantitative radiomic biomarkers and both genomic signatures and molecular phenotypes of gliomas [32].

dMRI is a widely used technique in the MRI assessment of brain gliomas [20], and clinical diffusion-weighted imaging (DWI) acquisitions with a maximum b-value of 1000 s/mm^2^ are included in the EORTC-NBTS consensus recommendations [22]. The dMRI-derived apparent diffusion coefficient (ADC), or mean diffusivity (MD), is largely considered an indirect measure of tumor cellularity, as proliferating tumor cells hinder the diffusion of extracellular water [34,35]. As such, ADC is inversely related to tumor cellularity and contributes to estimating tumor proliferation in LGG non-invasively, with minimum and mean diffusivity values higher than in high-grade gliomas [35]. Furthermore, quantitative ADC measurements have been recently reported to support the molecular subtyping of non-enhancing LGG in a clinical setting [36]. ADC values obtained from standard clinical DWI with a simple, two-dimensional region-of-interest (ROI) quantification were lower in IDH wild-type than in IDH-mutant LGG, thus supporting the importance of the extraction of quantitative, *radiomic* metrics for meaningful dMRI analysis [36]. More recently, a pilot study from The Cancer Genome Atlas has demonstrated that ADC values obtained from clinical DWI correlated with survival in patients with IDH-mutant and IDH wild-type gliomas regardless of WHO grade, suggesting a potential usefulness of quantitative ADC estimates as a prognostic marker to enhance risk stratification in brain gliomas [37]. 

In LGG follow-up, dMRI may have a role besides other advanced MRI sequences such as PWI and MRS to detect foci of malignant transformation or to settle the differential diagnosis between post-irradiation changes versus tumor recurrence [20]. Furthermore, the estimation of water diffusion directionality by Diffusion Tensor Imaging (DTI)-derived advanced metrics may be considered a potential useful tool to aid in the delineation of tumor margins and the detection of brain tumor infiltration [38]. DTI has been shown to be promising in identifying the early effects of chemotherapy in LGG patients, preceding modifications on cMRI and volumetry, although larger studies are warranted to define its applicability in a clinical setting [39].

PWI quantifies changes associated with neoangiogenesis, which correlate with glioma malignancy [20,30]. The dynamic susceptibility contrast (DSC) PWI technique is the primary method used in clinics [23], and the DSC-derived rCBV is the most validated measure to predict grading, time to progression, and survival in LGG [20,40]. Furthermore, rCBV has recently been demonstrated as an accurate radiomic predictive measure of IDH mutation status [41]. Indeed, raised rCBV values in treatment-naïve LGG are associated with a distinct hypoxia/angiogenesis transcriptome signature found in IDH wild-type tumors [41]. Other PWI techniques, such as dynamic contrast-enhanced (DCE) MRI or arterial spin labeling (ASL), are currently under investigation to define their impact in the assessment of LGG in clinics [20,23,42]. In particular, ASL, which uses magnetically labeled arterial blood protons as an endogenous contrast, appears to be promising for the evaluation of IDH mutation status of low-grade astrocytomas in combination with DWI-derived parameters [43]. 

In the follow-up setting of LGG, the use of PWI, with particular regard to DSC-derived rCBV, is part of the clinical workup to identify malignant transformation and to distinguish therapy effects (pseudo-progression or radiation necrosis) from true tumor progression [20,23]. In this clinical scenario, an accurate and reproducible quantification of PWI parameters is crucial to avoid pitfalls of subjectivity, being aware that threshold values are not simply transferable between institutions and even different software for perfusion analysis [23]. Standardization of PWI methods within and across sites is strongly advocated to ensure their reproducibility and reliability in clinical practice [44,45]. 

Proton MR spectroscopy (^1^H-MRS) has been extensively used to detect and quantify a number of endogenous metabolic biomarkers in LGG [20,46]. In recent years, one of the most relevant advances of ^1^H-MRS has been the possibility to non-invasively detect in vivo the intratumoral accumulation of 2-hydroxyglutarate (2HG) in brain gliomas. As 2HG is produced by all known IDH-mutant enzymes, evaluation of 2HG abundance is an alternative indirect method for determining IDH status [47,48]. A recent meta-analysis has shown an excellent diagnostic performance of 2HG-MRS in the prediction of IDH mutant glioma, with pooled sensitivity and specificity of 95% and 91%, respectively [49]. Despite the current technical challenges of 2HG-MRS, these are promising data and encourage a wider adoption of this technique in clinics. Furthermore, the longitudinal evaluation of 2HG levels by MRS has been proven to be feasible to quantify and localize spatiotemporal changes of this metabolite, thus highlighting the potential of serial 2HG-MRS evaluation during treatment and follow-up [50,51]. In particular, the in-vivo longitudinal measurement of intratumoral 2HG levels could be critical to assess the pharmacodynamics of molecular drugs and ultimately the efficacy of targeted treatment, such as mutant-*IDH1* inhibitors in glioma patients [52]. 

Besides quantitative advanced MRI techniques, functional MRI (fMRI) and diffusion MR tractography have become an essential part of the pre-surgical and intraoperative workup of lower-grade gliomas [53]. Task-based fMRI and diffusion tensor imaging (DTI) tractography have proven to be valid and sensitive tools for localizing the distinct eloquent cortical areas and subcortical white matter fiber bundles near or inside a tumor, showing good accuracy when compared with intraoperative direct electrical stimulation (DES) [53,54]. Resting-state fMRI functional connectivity as well as new advanced HARDI (high angular resolution diffusion imaging) tractography methods are improving and reshaping the role of these advanced functional MRI techniques for surgery of gliomas [53], although larger studies are still warranted to encourage their wide clinical implementation in the near future. A promising application of functional imaging techniques in the assessment of cortical plasticity of motor and language functions in gliomas is currently a matter of investigation, especially to define how cognitive functional recovery or impairment is mirrored by specific imaging modifications, and to understand the association between longitudinal functional changes and progression of disease [55].

Positron emission tomography (PET) imaging reflects fundamental metabolic patterns in brain gliomas [56]. In particular, PET with radiolabeled amino acids such as [^11^C-methyl]-methionine (^11^C-MET), O-(2-[^18^F]-fluoroethyl)-L-tyrosine (^18^F-FET), and 3,4-dihydroxy-6-[^18^F]-fluoro-L-phenylalanine (^18^F-FDOPA) has been proven to have a moderately high diagnostic accuracy to discriminate high and low-grade gliomas, but the overlap between tumor subtypes hampers clear separation [57,58]. Nonetheless, a clear role as independent prognostic tool is still not demonstrated, since studies are few and with conflicting results [58]. Amino acid PET can be performed to detect aggressive disease foci in anatomical MRI findings suggestive of WHO grade II glioma, hence possibly guiding biopsy and tumor resection, as well as radiation dose boosting [59]. An association between IDH status and amino acid PET parameters has been recently reported in LGG, as the IDH-1/2 wild-type lesions have greater metabolic activity than IDH1/2 mutated lower-grade gliomas in terms of the SUVmax and SUV ratio [60], even if these results need confirmation in larger studies. 

In the longitudinal assessment of LGG, amino acid PET can be performed in cases in which cMRI and aMRI are not conclusive, as it can be helpful for the metabolic detection of malignant transformation, as well as for the differentiation between treatment-related changes and true progression with high sensitivity and specificity [59]. However, the current scarce availability of amino acid PET, the use of ionizing radiations and, only for ^11^C-MET, the need for local access to a cyclotron, still prevent wide clinical use of PET imaging in LGG.

## 4. Role of Surgery

Although surgery plays a central role in the management of LGG patients today, its value has been debated for many years [21,61,62].

Numerous studies have recently demonstrated that the maximal safe extent of tumoral resection (EOR) is the first-line treatment resulting in better survival [21,63,64,65,66,67,68,69,70,71,72,73,74,75,76,77,78]. In the last decade, the qualitative and subjective descriptions of EOR as “gross total resection”, “near total resection”, and “subtotal resection”, are being replaced by precise and objective estimation based on the volume of residual tumor according to the following formula: “EOR = preoperative tumor volume − postoperative tumor volume/preoperative tumor volume” [21,66]. The methodological shift in tumor volume estimation has made surgical investigations more comparable to each other and independent of the surgeon’s subjective evaluation (Table 2).

The main aim in LGG surgery is to preserve functional integrity with maximal tumor resection [61,70,76,79,80]. With regard to radical resection in LGG, the critical areas that are of utmost importance in preserving quality-of-life functions and limiting functional damage postoperatively include the eloquent cortical areas and subcortical functional pathways. These are part of the complex motor and associative functions (i.e., reading, calculation, attention, language in its various sub-elaborations, vision, etc.) [21,63,66,70,81].

In order to optimize the management of these patients, personalized anatomo-functional planning and intraoperative strategy are needed. Modern neurosurgical oncologists rely on current methods and technology, which include frameless navigational systems, intra-operative imaging, navigated transcranial magnetic stimulation (nTMS), functional mapping, intraoperative neurophysiological monitoring, real-time neuropsychological testing (RTNT), and awake surgery [79,82,83,84].

The standard of care for LGG resection both at cortical and subcortical level involves DES (Direct Electrical Stimulation), which is used for both brain mapping and for monitoring neurologic performance, often in Awake Setting [82,85]. With regard to LGG close to or involving the motor pathways, it has been shown that there are lower risks of permanent postoperative deficits and higher EOR for lesions in eloquent areas when surgery is associated with intra-operative neurophysiological monitoring [86]. The gold standard for cerebral brain mapping is represented by awake surgery, considering that it is the only method that permits a real-time direct identification of neural networks [81,87]. In several studies, surgery based on awake mapping and real-time neuropsychological testing (RTNT) showed higher EOR and preservation of quality of life for LGGs involving both language and extra-language functional networks [81,82,87]. The currently available evidence supports attempting a gross-total resection if safe and feasible, but the strength of this recommendation must be prospectively validated [63]. Although the maximal safe resection remains the key element in the treatment of LGG, there is still no general consensus in literature regarding a minimum EOR cut-off value related to an effective survival benefit. Furthermore, the impact of the new 2016 WHO molecular subtypes, among the EOR classes, is still poorly investigated and the optimal postoperative treatment remains disputed, especially when a radical resection is not functionally possible [65,69]. Recently, Kavouridis et al. [69] demonstrated that the prognosis is influenced by minimal volumetric differences among the different molecular classes. More specifically, in subtypes of IDH-mutant and IDH-wildtype astrocytoma a residual tumor difference on only 1 cm^3^ influences survival. Otherwise, in oligodendroglioma patients, postoperative residuals impact on survival when exceeding 8 cm^3^.

Overall, these evidences suggest that postoperative clinical trials assessing the efficacy of adjuvant therapy for LGG should be stratified by molecular subtype and EOR. Future multicenter studies are required to determine the EOR cut-off value, stratified by the molecular class, which could benefit from adjuvant treatments optimizing the postoperative management of LGG.

## 5. Role of Radiotherapy 

In patients with LGG, the role of radiotherapy, either alone or in combination with chemotherapy, has long been debated as to its ability to answer questions that are still open and limit the clinical decision-making [21,100]. Current clinical practice has been derived from results of studies (see Table 3) designed many years ago, and therefore conducted with an old pathological classification of glioma, before the introduction of latest biomarkers and, in most of those clinical trials, when modern radiotherapy techniques such as Intensity Modulated Radiotherapy were not available [100]. As a consequence, clinical studies carried out over the last decades have not yet established the optimal use of radiotherapy in patients with LGG, and, mostly, the best timing to use ionizing radiations, their optimal dose, the most active concomitant and sequential chemotherapy still represent unanswered questions. 

Unfortunately, in patients with LGG, surgery alone does not have a curative function, and, to improve life expectancy, radiotherapy represents an important active treatment [21]. The first question concerning the use of radiotherapy in the postoperative setting is about its best timing-early, straight after surgery, or later, upon recurrence of the disease. Considering a balance between improving tumor control by postponing the recurrence, and limiting the side effects due to radiotherapy, both early and delayed treatments could have a rationale [106,107]. The EORTC 22845 randomized trial, initiated in 1986, has been designed to fulfil this question [102]. Early radiotherapy and salvage radiotherapy at the time of progression (both 54 Gy in six weeks) were studied in 314 randomized patients (patients were stratified based on histology as follows: astrocytoma versus oligodendroglioma or oligoastrocytoma). Despite differences recorded in median progression-free survival (PFS) (5.3 years in early versus 3.4 in delayed treatments, *p* < 0.0001) and a low incidence of seizure in patients treated straight after surgery, median overall survival (OS) was similar in two groups (7.4 and 7.2 years, respectively). Moreover, the authors did not record the quality of life and side effects related to radiotherapy, and only concluded that progression-free survival was better in early treated patients, whereas OS was not affected by early or delayed RT. In the following years, the lack of OS benefit recorded in this trial justified the choice to defer in selected patients with low-grade glioma and favorable clinical prognostic factors the start of radiotherapy until disease progression [108]. These patients were followed-up and treated with salvage radiotherapy only upon recurrence of the disease. In 2016, results of the RTOG 9802, a randomized trial designed to assess the use of sequential chemotherapy following radiotherapy, showed the same results as the EORTC 22845 trial [104]. Other than two interventional arms, the study was planned with an observational arm. In that study, the group of patients treated with postoperative radiotherapy had the same OS as the observational arm, where radiotherapy had a salvage attempt. Preoperative tumor diameter greater than 4 cm, residual disease ≥ 1 cm, and astrocytoma/oligoastrocytoma histology were associated with an increased risk of recurrence in patients managed with postoperative follow-up [109].

Currently, for patients with LGG undergoing gross tumor resection and IDH mut, initial observation after surgery rather than postoperative treatment can be preferred. In these patients, delaying radiotherapy does not impact on overall survival and prevents the onset of adverse events. The lack of patient stratification by biomarkers and the use of the outdated radiotherapy technique represent the main limits of the published studies, and future guidelines require further trials. 

Beyond the best timing, there is the choice of the most appropriate dose and fractionation of affordable radiotherapy. The first trial studying two different doses of ionizing radiations was conducted by the EORTC between 1986 and 1995 (even in this case, at a time when neuroradiology, radiotherapy, and patient assessment were different from today) [101]. Three hundred seventy-nine patients with low-grade gliomas (pilocytic astrocytomas, astrocytomas, oligodendrogliomas, and oligoastrocytomas) were randomized in two arms: 45 Gy delivered in 25 fractions and 59.4 in 33. After a median follow-up of six years, OS was 58% and 59% in the 45 Gy and the 59.4 Gy arms, respectively, whereas PFS was 47% and 50%. Differences recorded in OS and PFS were not statistically significant. Moreover, even differences in long-term sequelae were not different.

The trial sponsored by NCCTG/RTOG/ECOG aiming to assess the best dose of radiotherapy was conducted between 1986 and 1995 [103]. Two hundred and three patients with diffuse low-grade glioma (astrocytoma, oligoastrocytoma, and oligodendroglioma) were randomized to receive 50.4 Gy/28 fractions versus 64.8 Gy/36 fractions. In this trial, likewise, no differences were detected in OS and PFS. A secondary analysis revealed that only 5.3% of patients deteriorated after five years.

Considering the lack of results derived from recent clinical trials that considered the more recent WHO classification of LGG, performed with state of the art radiotherapy (and not with the radiotherapy performed 30 years ago) and with modern radiological imaging to identify the clinical target volume, currently, the dose of radiotherapy delivered to patients with LGG ranges between 45 and 54 Gy in 1.8/2 Gy fractions (it is a reasonable compromise between attempting to achieve tumor control and avoiding neurological side effects).

Once established that in patients with LGG a higher dose of radiotherapy did not improve OS or PFS, as it can on the contrary lead to a higher risk of neurotoxicity, several studies were planned to assess the introduction of concomitant or sequential chemotherapy to improve the efficacy of ionizing radiations and their therapeutic index. Between 1998 and 2002, the phase 3 study RTOG 9802 randomized 251 high-risk low-grade glioma patients (grade 2 astrocytoma, oligodendroglioma, or oligoastrocytoma) to receive 50 Gy alone or the same radiotherapy dose plus 12 cycles of CT with PCV (Procarbazine, Lomustine, and Vincristine) [104]. Tumor histology consisted of diffuse astrocytoma, oligodendroglioma, and mixed oligoastrocytoma. After a median follow-up of 11.9 years, despite an increased incidence of hematologic toxicity, the combination arm showed an improvement of both PFS and OS (Median OS: 13.3 versus 7.8 years, respectively, *p* = 0.003). The survival benefit was considerable in all histology and results of this trial supported the use of PCV after radiotherapy. 

Several studies carried out in high-grade gliomas analyzed temozolomide to be delivered in association with radiotherapy [21,110,111]. The phase 2 study RTOG 0424 combined radiotherapy (54 Gy) with concomitant and sequential temozolomide [112]. Between 2005 and 2009, that study enrolled 129 high-risk diffuse low-grade glioma patients (oligodendroglioma, oligoastrocytoma and astrocytoma). In a preliminary analysis, the three-year OS rate was 73.1%, better than historical control (the period of recruitment, when biomolecular classification of low-grade glioma was not yet introduced in clinical practice, represents the limit of this study). However, in patients with LGG, there are currently no data from prospective clinical studies comparing the activity on OS and PFS of PCV regimen with temozolomide. Consequently, the choice of the agent to be considered is based on clinicians’ experience for any patient. 

## 6. Role of Chemotherapy and New Systemic Treatments

**Figure 3:**
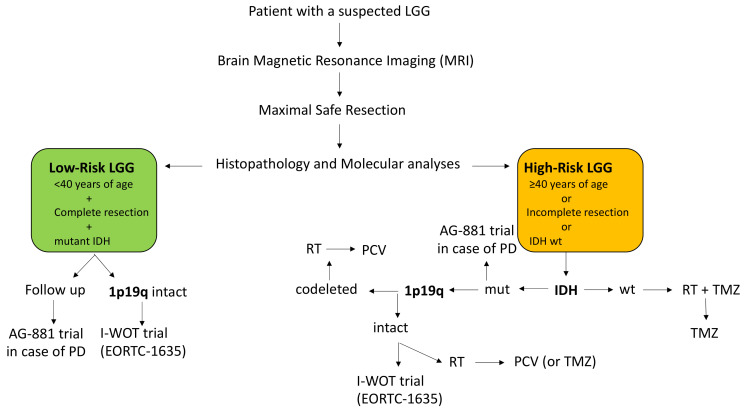
Current algorithm of LGG clinical management. LGG: low.grade glioma; RT: radiotherapy; TMZ: temozolomide; PCV: procarbazine, CCNU, Vincristine; IDH wt = IDH wild-type; PD: progressive disease.

Systemic treatments play an important role in the management of high-risk LGG (see Figure 3). The major current clinical trial is the RTOG 9802 trial [104] that compared radiotherapy exclusively versus radiotherapy and PCV regimen (procarbazine, CCNU and vincristine) for newly diagnosed high-risk LGG patients, defined as patients who have had incomplete tumor removal or as patients at least 40 years old (Table 4). In this trial, both progression-free survival and overall survival were significantly longer for patients receiving the combination of radiotherapy and PCV polychemotherapy with a tolerable safety profile. A recent molecular analysis on a fraction of patients from this trial [113] confirmed that patients with *IDH* mutated gliomas with or without 1p/19q codeletion benefited from the addition of PCV to radiotherapy, but suggested that patients with *IDH* wild-type astrocytomas may not benefit from this combination. Another phase III trial compared up-front chemotherapy alone by temozolomide versus radiotherapy alone for high-risk LGG [105]. If no difference between the two arms was observed on the whole cohort, radiotherapy seemed to be superior to chemotherapy for *IDH* mutated non-codeleted LGG on PFS. The methylation profile of the *IDH* mutated gliomas of this trial was then analyzed and seven CpGs of four DNA damage response genes (*MGMT, MLH3, RAD21,* and *SMC4*) might be predictive of PFS [114]. Moreover, the two *MGMT* CpGs identified (combined in a *MGMT* methylation score) might predict temozolomide benefits for *IDH* mutated patients regardless of codeletion status, suggesting a role for chemotherapy alone as initial treatment for a sub-group of patients with good prognosis and chemotherapy sensibility.

Finally, the RTOG 0424 phase II trial [112] evaluated the combination of radiotherapy and concomitant and adjuvant temozolomide for newly diagnosed high-risk LGG defined by the presence of at least three poor prognostic factors. In this trial, authors compared their results with those of an historical control cohort, suggesting that the combination could be beneficial for a subgroup of LGG displaying a particularly high risk of recurrence. However, these results should be confirmed in a randomized phase III trial. In summary, the combination of radiotherapy followed by PCV polychemotherapy is the current standard of care for newly diagnosed LGG patients who have undergone a subtotal resection or biopsy, or who are 40 years of age or older.

The I-WOT study by the EORTC brain tumor group (EORTC-1635-BTG) is an ongoing randomized, phase 3 study analyzing patients with *IDH* mutated 1p/19q intact lower grade glioma following resection, without a need for immediate post-operative treatment; the study will establish whether early adjuvant treatment with radiotherapy and adjuvant temozolomide in this clinically favorable group of patients will improve outcome compared to active surveillance (see Figure 3).

At relapse, treatment depends on first-line therapies. A second surgery or alkylating agents could be proposed. The main chemotherapy used at relapse is temozolomide because of its good blood-brain barrier penetration and its favorable safety profile [119,120]. Bevacizumab, commonly used for recurrent high-grade astrocytomas, was evaluated in the “TAVAREC” randomized phase II trial in association with temozolomide, but no survival benefit was observed [121]. Regarding the other potential drugs for LGG at recurrence, the use of everolimus was associated with disease stability in a phase II trial [122], while the development of immunotherapies like vaccines [123] could open new opportunities for LGG patients. By contrast, sunitinib [124] or imatinib [125,126,127] were insufficiently active. Moreover, ivosidenib, an inhibitor of mutant *IDH1*, showed interesting results in recurrent LGG with mutated *IDH* [128], and a randomized phase 3 study with the similar drug vorasidenib (AG-881) is currently ongoing. 

## 7. Epilepsy in Low-Grade Glioma

Seizures are frequently reported as the onset symptom in LGG, ranging from 25% to 80% with higher incidence in grade II [129,130]. Notably, the rate of attacks occurring more than three months before the diagnosis of a glioma is inversely related to the grade of the glioma, ranging up to 40% of patients with a LGG and representing an independent predictor of LGG when compared to high grade gliomas [130]. Seizure rate progresses over the course of tumor growth and reaches 90% incidence in diffuse LGG [131,132]. Risk factors for seizure are the IDH mutation and the location of LGG in superficial cortical, fronto-temporal, or insular regions [133,134]. A short seizure duration before surgery is associated with postoperative seizure control in LGG [135]. Since radiographic regression in LGG is slow, some authors proposed seizure frequency as a surrogate marker of tumor response in both trails and clinical setting [136,137]; we should note that seizures can be an early indicator of tumor progression, sometimes preceding tumor growth on MRI.

The extent of surgical resection is the main predictor of postoperative seizure control in LGG [135,138]. Radiation therapy and chemotherapeutic drugs for the treatment of LGG also contribute to seizure control [135,136]. Seizures arising from a brain tumor can be classified as symptomatic seizures with a focal onset, even if they manifest as a generalized seizure; therefore, antiepileptic drugs (AEDs) with available evidence for focal seizure control in the general population (such as levetiracetam, carbamazepine, and zonisamide), should be considered for the symptomatic treatment of LGG patients [139]. Among them, levetiracetam is a first choice in glioma patients for the lack of interaction with other drugs, its good tolerability, and its rapid titration [140,141]. Treatment can be initiated after the first seizure, while prophylactic treatment is not supported by solid evidence [142,143]. Rates of seizure freedom in 60–100% of cases were reported for levetiracetam, and in the range between 30% and 78% for pregabalin, valproic acid, topiramate, and oxcarbazepina in monotherapy [144,145,146,147,148]. 

When an add-on therapy is required, the combination of levetiracetam and valproic acid proved to be the most effective [144]. Favorable effects in add-on therapy were demonstrated, or emerged from preliminary data, also for the most recently marketed AEDs, such as lacosamide, lamotrigine, zonisamide, perampanel, and eslicarbazepine [146,149,150,151,152]. Lacosamide emerged as a valid alternative in focal seizures also in monotherapy [153,154], but solid evidence on large LGG population is still expected.

Non-cytochrome P450 enzyme-inducing AEDs such as levetiracetam and valproic acid should be preferred over enzyme-inducing ones such as phenytoin, carbamazepine, or older AEDs. Side effects are specific for each AED and include systemic (e.g., nausea, anorexia, thrombopenia, hair loss, weight gain) neurologic, and neurocognitive (e.g., diplopia, blurred vision, tremor, ataxia, somnolence, impaired cognitive performance) symptoms. To note, psychotic disorders are more commonly induced by levetiracetam [155] and LGG patients are particularly vulnerable because of the presence of tumor, the effects of surgery, and the concomitant oncological treatments.

Seizure and AED severely impact quality of life in LGG patients. Seizures can prevent several activities of daily living, including driving, swimming, and working, while side effects of AEDs contribute to the impairment of the quality of life [137]. AED withdrawal is controversial in patients with brain tumors and the risk of seizure recurrence should be counterbalanced by side effects of AEDs; the decision on withdrawal should be targeted to each individual patient [140,156]. 

A direct antitumor effect or an enhancement of antineoplastic drugs action has been advocated for some AEDs (i.e., valproic acrid, levetiracetam, perampanel, brivaracetam, and lacosamide) and some common pathways between epileptogenicity and glioma oncogenesis have been identified [150,157,158], but prospective clinical studies focusing on anticancer activity of AEDs are still lacking. 

## 8. Conclusions

Questions regarding the optimum management of LGG persist although molecular characterization, diagnosis, and treatment are evolving. In the recent WHO classification of gliomas, the genetic profile of LGG was integrated with histological features providing subclasses with different biological behavior and outcome. Analysis of tumor activity using new neuroradiological tools may improve the diagnostic and therapeutic strategies for these tumors. Surgery plays a central role in delaying tumor progression and malignant transformation, and maximal safe surgical resection is recommended. Following surgical resection, radiotherapy and alkylating agents should be used for “high-risk” patients. However, according to recent studies, specific post-surgical treatment should be personalized and based on *IDH* mutational status and 1p/19q codeletion. Other clinical trials are ongoing to better understand the optimal strategy. New targeted therapies and immunotherapy involving the mutated IDH protein could improve the outcome in selected cases.

## Figures and Tables

**Table 1:** Molecular classification of diffuse low-grade gliomas (LGGs).

	DIFFUSE LGGs				
Features	Diffuse Astrocytoma *IDH* Mutant	Oligodendroglioma *IDH* Mutant and 1p/19q Codeleted	Diffuse Astrocytoma *IDH*-wt		
			“early stage” GBM	diffuse glioma NEC	Diffuse astrocytoma *IDH*-wt
*IDH* status	*IDH* mutation	*IDH* mutation	*IDH* wt	*IDH* wt	*IDH* wt
1p/19q codeletion	absent	present	absent	absent	absent
genetic alterations	*ATRX* mutation	*ATRX* wt	*EGFR* or *CDK4* or *MDM4* ampl, *pTERT* mut, *CDKN2A* del, ch 7 gains, ch 10 losses	*BRAF* V600E mut, *FGFR1* or *MYB* or *MYBL1* alterations	Absence of K27M mutation in *H3F3A* or *HIST1B* or *HIST1C*
	*TP53* mutation	*TP53* wt			other genetic alterations not investigated or absent
Prognosis	intermediate	good	bad	good for tumors with *MYB* or *MYBL1* alterations	

LGGs: low grade gliomas; Wt: wild type; GBM: glioblastoma; NEC: not elsewhere classified.

**Table 2:** Literature review of volumetric studies in low-grade gliomas.

Study	Year	N. of pts	Tumor Type	Extent of resection	5-Year Survival
Claus et al [67]	2005	156	Oligodendroglioma 95; Astrocytoma 35; Mixed 26	100% (56)	98.2%
				<100% (100)	92%
Smith et al [78]	2007	216	Astrocytoma 93; Oligodendroglioma 91; Mixed 32	100% (75)	98%
				90–99% (26)	97%
				70–89% (55)	nd
				41–69% (39)	nd
				0–40% (21)	nd
Sanai et al [76]	2010	70	“Grade II glioma”	91–100% (14)	100%
				≤90% (56)	84%
Skrap et al ^a^ [77]	2012	53	Astrocytomas with gemistocytic foci: 2; Fibrillar astrocytomas: 34; Oligoastrocytomas: 10; Oligodendrogliomas: 7	≥90% (22) ^**^	92%
				70–89% (30)	82%
				<70% (14)	57%
Ius et al [70]	2012	190	Fibrillary astyrocytoma 98; Oligoastrocytoma 34; Oligodendroglioma 58	≥90% (91)	93%
				70–89% (69)	84%
				<70% (30)	41%
Nitta et al [74]	2013	153	Astrocytoma 49; Oligoastrocytoma 45; Oligodendroglioma 59	≥90% (94)	98.4%
				<90% (59)	89.7%
Capelle et al [64]	2013	674	“Grade II glioma”	100% (80)	100%
				50–99% (418)	88%
				<50% (431)	77%
Majchrzak et al [88]	2012	68	Astrocytoma 46; Oligodendroglioma 5; Mixed 17	≥95% (21)	100%
				85–95% (13)	100%
				<85% (34)	81% ^*^
Snyder et al [89]	2014	93	Oligodendroglioma 93	≥90% (42)	90%
				<90% (51)	87%
Coburger et al [90]	2016	288	Astrocytoma 173; Oligodendroglioma 52; Mixed 63	100% (138)	OS: 302 months
				<100% (149)	Failed GTR, OS: 171 months Intended STR, OS: 162 months
Jungk et al [91]	2016	46	Astrocytoma 46	100% (10)	nd
				90–99% (11)	
				41–89% (14)	
				<40% (7)	
				nd (4)	
Roelz et al [92]	2016	49	Astrocytoma 18; Oligodendroglioma 12; Mixed 19	RTV < 15 cm^3^ (27)	96%
				RTV > 15 cm^3^ (22)	64%
Eseonu et al [93]	2017	25	“Grade II glioma”	≥90% (nd)	100%
				<90% (nd)	80%
Eseonu et al [94]	2017	109	Astrocytoma 73; Oligodendroglioma 36	100% (34)	95%
				90–99% (25)	92%
				70–89% (24)	82%
				<70% (26)	76%
Patel et al [95]	2018	74	Astrocytoma 43; Oligodendroglioma 19; Mixed 12	IDHmt (73.3%) ^+++^	95.2% ^++++^
				IDHwt (27.8%) ^+++^	55.0% ^++++^
Wijnenga et al [96]	2018	228	Oligodendroglioma (IDHmt, 1p/19q codeleted) 93; Astrocytoma IDHmt 112; Astrocytoma IDHwt 23	100% (35)	93.75%
				95–99% (14)	90.6%
				90–94% (22)	84.4%
				41–89% (90)	87.5%
				0–40% (67)	56.25%
Hameed et al [97]	2018	120	Diffuse astrocytoma, IDH1 mutant 56; Diffuse astrocytoma, IDH1 wild-type 22; Diffuse astrocytoma, NOS 5; Oligodendroglioma, IDH1 mutant & 1p/19q-codeleted 25; Oligodendroglioma, NOS 7;Oligoastrocytoma, NOS 5	≥90% (93)	Mean OS 68.51 months
				<90% (27)	Median OS 49.80 months
Morshed et al ^b^ [98]	2018	26	Diffuse astrocytoma, IDH1 mutant 5; Diffuse astrocytoma, IDH1 wild-type 7; Oligodendroglioma, IDH1 mutant & 1p/19q-codeleted 13; Oligoastrocytoma, NOS 1	100% (8)	nd
				70–99% (7)	
				<70% (11)	
Ius et al [71]	2018	146	Diffuse astrocytoma, IDH mutant 81; Diffuse astrocytoma, IDH wild-type 8; Oligodendroglioma, IDH1 mutant & 1p/19q-codeleted 57	86%	74%
Bo et al [99]	2019	47	Diffuse astrocytoma, IDH1 mutant 20; Diffuse astrocytoma, IDH1 wild-type 7; Oligodendroglioma, IDH1 mutant & 1p/19q-codeleted 19; Oligodendroglioma, NOS 1	100% (14) 90–99% (14) <90% (19)	significantly better OS with postoperative tumor remnant of less than 10 ml (estimated 5-year survival 94% vs 53%, *p* = 0.03).
Cesselli et al [68]	2019	241	Diffuse astrocytoma, IDH1 mutant 20; Diffuse astrocytoma, IDH1 wild-type 7; Oligodendroglioma, IDH1 mutant & 1p/19q-codeleted 19;		

Pts.: patients; IDHmt: *IDH* mutated; IDHwt: IDH wild-type; OS: Overall Survival; GTR: gross total resection; STR: subtotal resection; RTV: residual tumor volume; ^*^: OS for patients with EOR < 80%; ^**^: it comprises all study patients, including 13 high-grade gliomas (HGG); ^+++^: median EOR; ^++++^: 3-year OS; ^a^: only insular tumors; ^b^: all patients are older than 60.

**Table 3:** Leading studies evaluating the role of radiotherapy in LGG.

Trial	Treatments	Number of Patients	Median Overall Survival (Years)	Median PFS	5-Year OS (%)	5-Year PFS (%)
Karim et al. EORTC 22844 [101]	45 Gy in 25 ff	171	NA	NA	58	47
	59.4 Gy in 33 ff	172	NA	NA	59	50
Van den Bent et al. EORTC 22845 [102]	54 Gy in 30 ff	157	7.4	5.3	68	55
	Observation	157	7.2	3.4	66	35
Shaw et al. NCCT/RTOG/ECOG [103]	50.4 Gy in 33 ff	101	NA	NA	72	55
	64.8 Gy in 36 ff	102	NA	NA	64	52
Buckner et al. RTOG 9802 [104]	54 Gy in 30 ff	126	7.8	4.0 Years	63	44
	54 Gy in 30 ff + PCV × 6	125	13.3	10.4 Years	72	61
Baumert et al. EORTC 22033-26033 [105]	TMZ × 12 cycles	237	NR	39 months	NA	29
	50.4 Gy in 28 ff	240	NR	46 months	NA	40

ff: fractions; PFS: progression free survival; OS: overall survival; PCV: Procarbazine, CCNU and Vincristine; TMZ: temozolomide. NA: not available.

**Table 4:** Clinical trials evaluating systemic treatments for newly diagnosed LGG patients.

Clinical Trial	Phase	Patients	Arm(s)	Results
**RTOG 9802** [104]	III	≥40 years or subtotal resection or biopsy	RT versus RT-PCV	RT-PCV > RT for OS and PFS
**EORTC 22033-26033** [105]	III	>40 years or progressive disease or tumor > 5cm or crossing midline or neurological symptoms	RT versus TMZ	No difference for PFS (all patients) Subgroup analyses: *IDHm*/non-codel: RT > TMZ for PFS *IDHm*/codel and *IDHwt*: no difference
**RTOG 0424** [112]	II	3 or more: ≥40 years, astrocytoma, bihemispherical tumor, preoperative tumor size ≥ 6 cm, preoperative neurological function status > 1	RT-TMZ	5-year OS rate: 60.9% Median OS: 8.2 years (95%CI: 5.6–9.1)
**Eyre et al.** [115]	II	Incomplete surgical resection	RT versus RT-CCNU	No difference between treatment arms Median OS (all patients): 4.45 years
**Ruda et al.** [116]	II	Incomplete surgical resection or biopsy or progressive disease	TMZ alone	Median PFS: 3.4 years (95%CI: 2.2–4.3) Median OS: 9.2 years (95%CI: 8.2–11.9)
**Wahl et al.** [117]	II	Gross residual disease after resection	TMZ alone	Median PFS: 4.2 years (95%CI: 3.0–5.0)Median OS: 9.7 years (95%CI: 7.2–11.3)
**Kaloshi et al.** [118]	II	Progressive disease, refractory epilepsy, neurological deficit	CCNU alone	Median PFS: 27.8 months (95%CI: 21.2–59.6) 5-year OS rate: 71%
**Kesari et al.** [119]	II	Oligodendroglioma and oligoastrocytoma with a MIB-1 index > 5% or recurrent LGG	TMZ alone	5-year OS rate: 73% 5-year PFS rate: 34%

RT: radiotherapy; PCV: procarbazine, CCNU, Vincristine; OS: Overall Survival; PFS: Progression-Free Survival; TMZ: temozolomide; *IDHm*: IDH mutated; *IDHwt*: *IDH* wild-type; codel: codeleted; 95%CI: 95% confidence Interval; LGG: low-grade glioma.
